# Prognostic features of biochemical recurrence of prostate cancer following radical prostatectomy based on diffusion kurtosis imaging

**DOI:** 10.1186/s40644-026-00997-y

**Published:** 2026-01-28

**Authors:** Li-Peng Lin, Xian-Wen Cheng, Juan Chen, Shu-Yi Li, Jie Bian, Jin-Kun He, Di-Min Liu, Qing-Yu Liu, Kun-Peng Zhou

**Affiliations:** 1 Department of Radiology, The Seventh Affiliated Hospital, Sun Yat-sen University, No.628, Zhenyuan Road, Xinhu Street, Guangming District, Shenzhen, Guangdong 518107 P. R. China; 2 Medical Imaging Center, Shenzhen Pingle Orthopedic Hopital (Shenzhen Pingshan Traditional Chinese Medicine Hospital), Shenzhen, 518118 P. R. China; 3 Department of Radiology, The Second Hospital of Dalian Medical University, Dalian, 116027 P. R. China

**Keywords:** Prostate cancer, Biochemical recurrence, Diffusion kurtosis imaging, Magnetic resonance imaging, Radical prostatectomy

## Abstract

**Objectives:**

To evaluate characteristics of diffusion kurtosis imaging (DKI), baseline prostate-specific antigen (PSA) and postoperative histopathological findings associated with biochemical recurrence (BCR) of prostate cancer (PCa) after radical prostatectomy (RP).

**Materials & Methods:**

Totally, 44 BCR patients and 72 non-BCR patients who underwent RP from April 2019 to March 2021 were retrospective collected. Baseline PSA, parameters of DKI and histopathological characteristics of the patients were evaluated. Continuous variables were compared by independent sample t-test, and categorical variables were compared by Chi-square test and Fisher's exact test. Cox regression analysis was used to analyze the association between relevant characteristics and BCR. p<0.05 was considered statistically significant.

**Results:**

Results of univariate Cox regression analysis showed that baseline T-PSA (p<0.001), F-PSA (p=0.034), F/T-PSA (p<0.001), mean diffusivity (MD) (p=0.002), mean kurtosis (MK) (p<0.001) and apparent diffusion coefficient (ADC) (p=0.021) values, ISUP grade group 4 or 5 (p<0.001), positive extraprostatic extension (EPE) (p<0.001), positive perineural invasion (p=0.041), Positive surgical margin (p=0.001) and positive lymphovascular invasion (p<0.001) were correlated with BCR. However, results of multivariate Cox regression analysis showed that baseline T-PSA (HR=10.579; 95% CI, 4.405–27.670; p<0.001), MK value (HR=3.034; 95% CI, 1.809–5.089; p<0.001), ISUP grade group 4 or 5 (HR=3.121; 95% CI, 1.352–7.209; p=0.008), positive EPE (HR=2.219; 95% CI, 1.058–4.651; p=0.035) and positive surgical margin (HR=2.816; 95% CI, 1.585–4.845; p=0.025) were independent risk factors for BCR.

**Conclusion:**

MK value, as well as baseline F-PSA, ISUP grade group 4 or 5, positive EPE and surgical margin were associated with BCR of PCa after RP.

**Supplementary Information:**

The online version contains supplementary material available at 10.1186/s40644-026-00997-y.

## Key points

**Question:**

The association between preoperative diffusion kurtosis imaging and biochemical recurrence of prostate cancer after radical prostatectomy is unknown.

**Findings:**

Preoperative diffusion kurtosis imaging, as well as clinical and histopathological characteristics of prostate cancer, were significantly associated with biochemical recurrence.

**Clinical relevance:**

Combining preoperative diffusion kurtosis imaging with baseline F-prostate-specific antigen and postoperative histopathological characteristics enables more precise risk stratification for biochemical recurrence after radical prostatectomy, thereby informing individualized surveillance planning and facilitating earlier detection of biochemical recurrence.

**Supplementary Information:**

The online version contains supplementary material available at 10.1186/s40644-026-00997-y.

## Introduction

Prostate cancer (PCa) is among the most common malignancies in men. With the widespread adoption of prostate-specific antigen (PSA) screening, detection rates have steadily increased [1]. Radical prostatectomy (RP) remains a first-line treatment for patients with localized or locally advanced disease; however, the 5-year risk of biochemical recurrence (BCR) after RP remains substantial (approximately 27%–52%) [2–4]. Notably, BCR often precedes metastatic progression and PCa–specific mortality. Studies have shown that timely salvage radiotherapy combined with androgen deprivation therapy for patients with BCR can improve overall survival and reduce the incidence of metastasis and related mortality [5, 6]. Consequently, accurate identification of patients at high risk of BCR is critical for optimizing individualized treatment and surveillance strategies.

Existing BCR prediction models are primarily based on preoperative baseline PSA and postoperative histopathological characteristics. Higher preoperative baseline PSA, pathological extraprostatic extension (EPE), seminal vesicle invasion, positive surgical margin, and Gleason pattern ≥ 8 are all associated with increased BCR risk [7, 8]. Nevertheless, most of these models do not incorporate multiparametric magnetic resonance imaging (mpMRI), which may limit their ability to capture tumor biological heterogeneity and microstructural characteristics.

With continuous refinements in the Prostate Imaging Reporting and Data System (PI-RADS), mpMRI has achieved broader clinical adoption and improved inter-reader consistency [9]. Diffusion-weighted imaging (DWI), a core prostate mpMRI sequence, plays a pivotal role in the detection and risk assessment of PCa; yet its characterization of complex tissue microstructure is constrained by the non-Gaussian behavior of water diffusion. Diffusion kurtosis imaging (DKI), an advanced sequence of DWI, can capture non-Gaussian diffusion and thus more comprehensively reflect tumor microarchitecture [10]. Prior studies suggested that DKI may enhance the detection of clinically significant PCa and aid in aggressiveness assessment [11–15]. However, to our knowledge, evidence on using DKI to predict BCR after RP is currently lacking.

Against this background, the aim of the present study was to explore the preoperative baseline PSA and postoperative histopathological features associated with BCR after RP, and more importantly to clarify the relationship between preoperative DKI-related parameters and BCR.

## Materials and methods

### Patients

The study was approved by *the ethics committee of the Second Affiliated Hospital of Dalian Medical University* (PR/AG- 342/2020, approved on 3 February 2020). The study protocols were conducted in accordance with the tenets of the Declaration of Helsinki. From April 2019 to March 2021, we retrospective enrolled 143 patients who underwent RP at our institution and had pathologically confirmed PCa. All patients underwent preoperative mpMRI. Exclusion criteria were as follows: [1] receipt of radiotherapy, chemotherapy, or androgen-deprivation therapy prior to mpMRI (*n* = 4); [2] mpMRI with substantial artifacts precluding reliable assessment (*n* = 3); [3] non-compliant follow-up after RP: PSA monitoring intervals > 6 months during the first 3 postoperative years or > 12 months during postoperative years 4 ~ 5 (*n* = 11) [16]; [4] missing follow-up information (*n* = 9). A total of 116 patients were enrolled in the final analysis, including 44 BCR group patients and 72 non-BCR group patients. BCR was defined as a postoperative decline of PSA below the assay’s lower limit of detection followed by two consecutive PSA measurements > 0.2 ng/mL [16]. BCR-free survival was calculated from the date of RP to the date of BCR or the last follow-up, whichever occurred first. Patients without BCR were censored at 5 years postoperatively.

### MRI techniques

All patients were performed on a 3.0-T MR system (GE Discovery MR750w, GE Healthcare) using an eight-channel phased-array external coil. In accordance with PI-RADS version 2.1 [9], the conventional sequences comprised T_2_-weighted imaging (T_2_WI), T_1_-weighted imaging (T_1_WI), diffusion-weighted imaging (DWI), and dynamic contrast-enhanced MRI (DCE-MRI). In our study, DKI, as an advanced DWI sequence employing multiple b-values, utilized 15 gradient directions and five b values (b values [number of excitations], 0 [1], 500 [2], 1000 [4], 1500 [6], 2000 [8] s/mm²). Another acquisition parameters of DKI were as follows: repetition time/echo time (TR/TE), 3800/87.0 ms; slice thickness, 3 mm; interslice gap, 0 mm; field of view (FOV), 24 × 24 cm; matrix, 256 × 224 [11]. Additional MRI technique details are provided in Table 1.

**Table 1:** Acquisition parameters of the multiparametic MRI protocol

Sequence	Imaging plane	TR/TE (ms)	Slice/Gap (mm)	FOV (cm^2^)	Matrix	b (s/mm^2^)
T_2_WI	Oblique axial, Oblique coronal, sagittal	3200 ~ 3500/95.0	3.0/0	20 × 20	256 × 256	-
T_1_WI	Oblique axial	767/9.6	3.0/0	24 × 24	256 × 224	-
DWI	Oblique axial	3500/73.0	3.0/0	24 × 24	256 × 224	0,2000
DCE-MRI	Oblique axial	3.8/1.4	3.0/0	24 × 24	256 × 224	
DKI	Oblique axial	3800/87.0	3.0/0	24 × 24	256 × 224	0, 500, 1000, 1500, 2000

DWI, diffusion weighted imaging; DCE-MRI, dynamic contrast-enhanced imaging MRI; DKI, diffusion kurtosis imaging; TR, repetition time; TE, echo time; FOV, field of view

### Imaging and quantitative data analysis

All prostate MRI were evaluated in accordance with the criteria of PI-RADS version 2.1 [9]. Two radiologists (DM. L., 11 years of abdominal imaging experience; and KP. Z., 8 years of abdominal imaging experience) jointly evaluated the images while blinded to laboratory results and ancillary imaging (e.g., ultrasonography). In the event of discrepancy in the diagnostic conclusions, a senior radiologist (QY. L., more than 20 years of abdominal imaging experience) adjudicated to reach a final consensus.

For all included participants, the reconstruction and quantitative analysis were carried out on GE AW 4.6 workstation. Firstly, region of interest (ROI) was drawn along the lesion outline at the largest axial slice on apparent diffusion coefficient (ADC) map. The ROI was copied onto the related parameter maps of DKI for quantitative measurements, subsequently. If a patient has multiple lesions (≥ 2 lesions), the one with the largest volume is typically selected as the target lesion for quantitative measurements (MK and MD values). All processing steps were standardized across cases.

### Histopathological analysis

Histopathological assessment of each RP specimen was jointly conducted by two pathologists (both K. G. and J. X., with more than 10 years of experience in Histopathological diagnosis), who blind to the prostate mpMRI outcomes. Discrepancies between the evaluations were reconciled through consensus. Histopathological evaluation included International Society of Urological Pathology (ISUP) grade group, EPE, lymphovascular invasion, perineural invasion and surgical margin. Reporting for RP specimen was performed according to ISUP protocol [17].

### Statistical analysis

Continuous variables were first assessed for normality using the Shapiro–Wilk test and for homogeneity of variances using the Levene test. For variables satisfying these assumptions, independent sample *t*-test was used to compare non-BCR vs. BCR groups. categorical variables were compared using Chi-square test or Fisher’s exact test when appropriate. DKI parameters were likewise compared between groups using independent sample *t*-test. Potential multicollinearity among covariates was examined through linear-regression diagnostics by computing the variance inflation factor. The proportional hazards assumption for the Cox model was evaluated using Schoenfeld residuals. Thereafter, multivariable Cox proportional hazards regression was performed, and results are presented as hazard ratio (HR) with 95% confidence intervals and P values. Multicollinearity among variables was assessed using the variance inflation factor (VIF). If multicollinearity was identified, collinear variables were entered into the model separately, and model performance was evaluated for each specification. The variable yielding the best model performance was then retained for subsequent analyses. To mitigate overfitting, a penalized Cox regression model (Ridge penalty) combined with cross-validation was applied to derive a robust model. A Kaplan-Meier analysis was completed for a combination of selected variables in the parsimonious model, and the log-rank test was used for comparison between groups. All tests were two-sided with statistical significance set at *p* < 0.05. Analyses were conducted using SPSS software (v. 27.0, SPSS, Chicago, IL) and GraphPad Prism (V.10.1; GraphPad Software, San Diego, USA).

## Results

### Patient characteristics

The study included 44 BCR group patients (age 74.16 ± 5.46 years) and 72 non-BCR group patients (age 73.18 ± 6.28 years). The median follow-up time was 18.91 months (range, 3.71–58.97 months) for patients with BCR and 59.70 months (range, 57.46–61.34 months) for patients without BCR. The mean time to BCR was 23.92 ± 15.84 months. Compared with non-BCR group, BCR group had significantly higher baseline total PSA (T-PSA) (12.17 ± 3.05 ng/mL vs. 17.32 ± 5.06 ng/mL, F = 17.432, t=-6.845, *p* < 0.001) and significantly lower baseline free-to-total PSA (F/T-PSA) (0.198 ± 0.051 vs. 0.155 ± 0.031, F = 8.932, t = 5.033, *p* < 0.001). Details showed in Table 2. Results of univariable Cox analysis showed that higher baseline T-PSA (HR: 28.821; 95% CI: 10.763 ~ 77.182; *p* < 0.001), higher baseline F-PSA (HR: 2.131; 95% CI: 1.057 ~ 4.297; *p* = 0.034) and lower baseline F/T-PSA (HR: 0.106; 95% CI: 0.042 ~ 0.267; *p* < 0.001) were significantly associated with higher risk of BCR, with specific results provided in Table 3.

**Table 2:** Clinical, MRI and histopathological characteristics according to cohort

Description	Non-BCR (*n* = 72)	BCR (*n* = 44)	*p*
Age (year)	73.18 ± 6.28	74.16 ± 5.46	0.394
Baseline T-PSA (ng/mL)	12.17 ± 3.05	17.32 ± 5.06	< 0.001
Baseline F-PSA (ng/mL)	2.38 ± 0.77	2.60 ± 0.61	0.114
Baseline F/T-PSA	0.198 ± 0.051	0.155 ± 0.031	< 0.001
PI-RADS category			
2	4 (5.6)	1 (2.3)	0.532
3	10 (13.9)	4 (9.1)	
4	26 (36.1)	14 (31.8)	
5	32 (44.4)	25 (56.8)	
Location			
PZ	48 (66.7)	27 (61.4)	0.704
TZ	24 (33.3)	17 (38.6)	
ISUP grade group			
1	13 (18.1)	3 (6.8)	0.012
2	29 (40.3)	11 (25.0)	
3	23 (31.9)	15 (34.1)	
4	6 (8.3)	13 (29.6)	
5	1 (1.4)	2 (4.5)	
RP EPE			
Positive	17 (23.6)	23 (52.3)	0.003
Negative	55 (76.4)	21 (47.7)	
RP perineural invasion			
Positive	34 (47.2)	29 (65.9)	0.077
Negative	38 (52.8)	15 (34.1)	
RP surgical margin			
Positive	9 (12.5)	15 (34.1)	0.011
Negative	63 (87.5)	29 (65.9)	
RP lymphovascular invasion			
Positive	6 (8.3)	16 (36.4)	< 0.001
Negative	66 (91.7)	28 (63.6)	
MK	0.548 ± 0.083	0.639 ± 0.065	< 0.001
MD (×10^− 3^mm^2^/s)	0.875 ± 0.123	0.797 ± 0.117	0.001
ADC (×10^− 3^mm^2^/s)	1.057 ± 0.171	0.968 ± 0.156	0.006

BCR, biochemical recurrence; T-PSA, total prostate specific antigen; F-PSA, free prostate specific antigen; PI-RADS, Prostate Imaging Reporting and Data System; TZ, transition zone; PZ, peripheral zone; ISUP, International Society of Urological Pathology; EPE, extraprostatic extension; RP, radical prostatectomy; MK, mean kurtosis; MD, mean diffusivity; ADC, apparent diffusion coefficient

**Table 3:** Cox regression analysis for biochemical recurrence in univariable and multivariable analyses

Description	Univariable Analysis		Multivariable Analysis	
	HR (95CI%)	*p*	HR	*p*
Age	1.156 (0. 695 ~ 1.922)	0.576	0.674 (0.351 ~ 1.296)	0.237
Baseline T-PSA	28.821 (10.763 ~ 77.182)	< 0.001	10.579 (4.045 ~ 27.670)	< 0.001
Baseline F-PSA	2.131 (1.057 ~ 4.297)	0.034		
Baseline F/T-PSA	0.106 (0.042 ~ 0.267)	< 0.001		
PI-RADS category				
2 ~ 4	Reference		Reference	
5	1.791 (0.706 ~ 4.546)	0.220	0.828 (0.283 ~ 2.423)	0.730
Location				
TZ	Reference		Reference	
PZ	1.115 (0.608 ~ 2.046)	0.725	1.341 (0.633 ~ 2.841)	0.444
ISUP grade group				
1 ~ 3	Reference		Reference	
4 ~ 5	4.667 (2.484 ~ 8.770)	< 0.001	3.121 (1.352 ~ 7.209)	0.008
RP EPE				
Negative	Reference		Reference	
Positive	2.826 (1.560 ~ 5.119)	< 0.001	2.219 (1.058 ~ 4.651)	0.035
RP perineural invasion				
Negative	Reference		Reference	
Positive	1.920 (1.029 ~ 3.583)	0.041	1.250 (0.566 ~ 2.765)	0.581
RP surgical margin				
Negative	Reference		Reference	
Positive	2.794 (1.493 ~ 5.230)	0.001	2.816 (1.585 ~ 4.845)	0.025
RP lymphovascular invasion				
Negative	Reference		Reference	
Positive	4.363 (2.342 ~ 8.129)	< 0.001	1.858 (0.721 ~ 4.791)	0.200
MK	3.368 (2.221 ~ 5.105)	< 0.001	3.034 (1.809 ~ 5.089)	< 0.001
MD	0.681 (0.535 ~ 0.868)	0.002	0.915 (0.665 ~ 1.258)	0.583
ADC	0.810 (0.677 ~ 0.968)	0.021	0.830 (0.670 ~ 1.028)	0.088

T-PSA, total prostate specific antigen; F-PSA, free prostate specific antigen; PI-RADS, Prostate Imaging Reporting and Data System; TZ, transition zone; PZ, peripheral zone; ISUP, International Society of Urological Pathology; EPE, extraprostatic extension; RP, radical prostatectomy; MK, mean kurtosis; MD, mean diffusivity; ADC, apparent diffusion coefficient

### Histopathological characteristics

Results of Chi-square test showed that significant between-group differences for RP ISUP grade group (*p* = 0.012), EPE (*p* = 0.003), surgical margin (*p* = 0.011), and lymphovascular invasion (*p* < 0.001). In univariable Cox analyses, RP ISUP grade group 4 or 5 (*p* < 0.001), positive EPE (*p* < 0.001), positive perineural invasion (*p* = 0.041), positive surgical margin (*p* = 0.001), and positive lymphovascular invasion (*p* < 0.001) were significantly associated with an increased risk of BCR, with RP ISUP grade group 4 or 5 showing the strongest association (HR = 4.667, 95% CI: 2.484–8.770; *p* < 0.001). Details showed in Tables 2 and 3.

### MRI characteristics

**Fig. 1:**
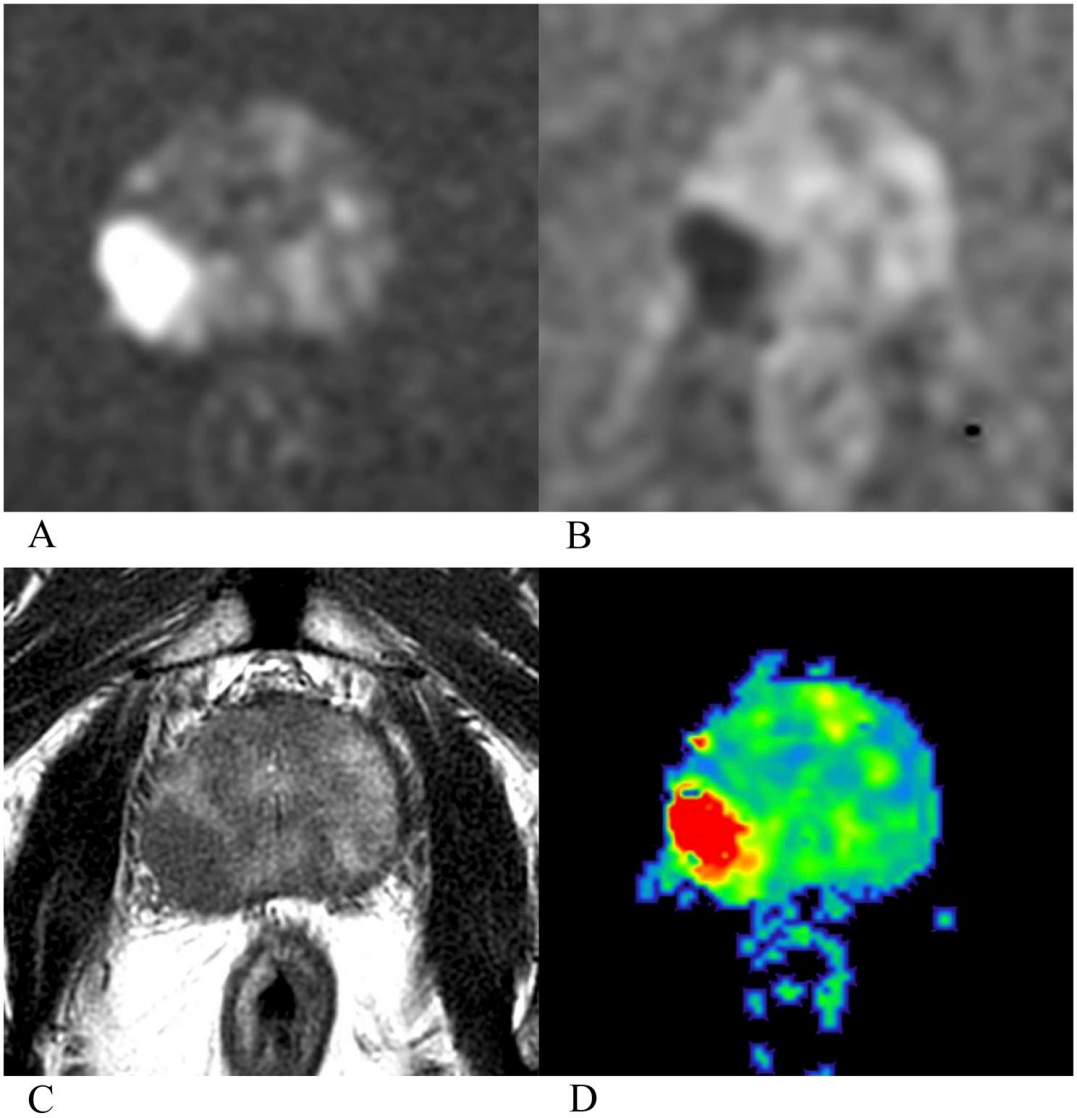
A 77-year-old International Society of Urological Pathology grade group 4 prostate cancer patient, with positive extraprostatic extension, positive perineural invasion, positive lymphovascular invasion and negative surgical margin, baseline T-PSA was 19.24 ng/ml, baseline F-PSA was 2.50 ng/ml, baseline F/T-PSA was 0.13. Biochemical recurrence was diagnosed at 13.5 months after radical prostatectomy. Preoperative multi-parametric MRI (**A**, diffusion weighted imaging; **B**, apparent diffusion coefficient map; **C**, T_2_-weighted imaging) showed that the PI-RADS 5 lesion was located in the right peripheral zone, and the mean kurtosis (MK) value was 0.704

**Fig. 2:**
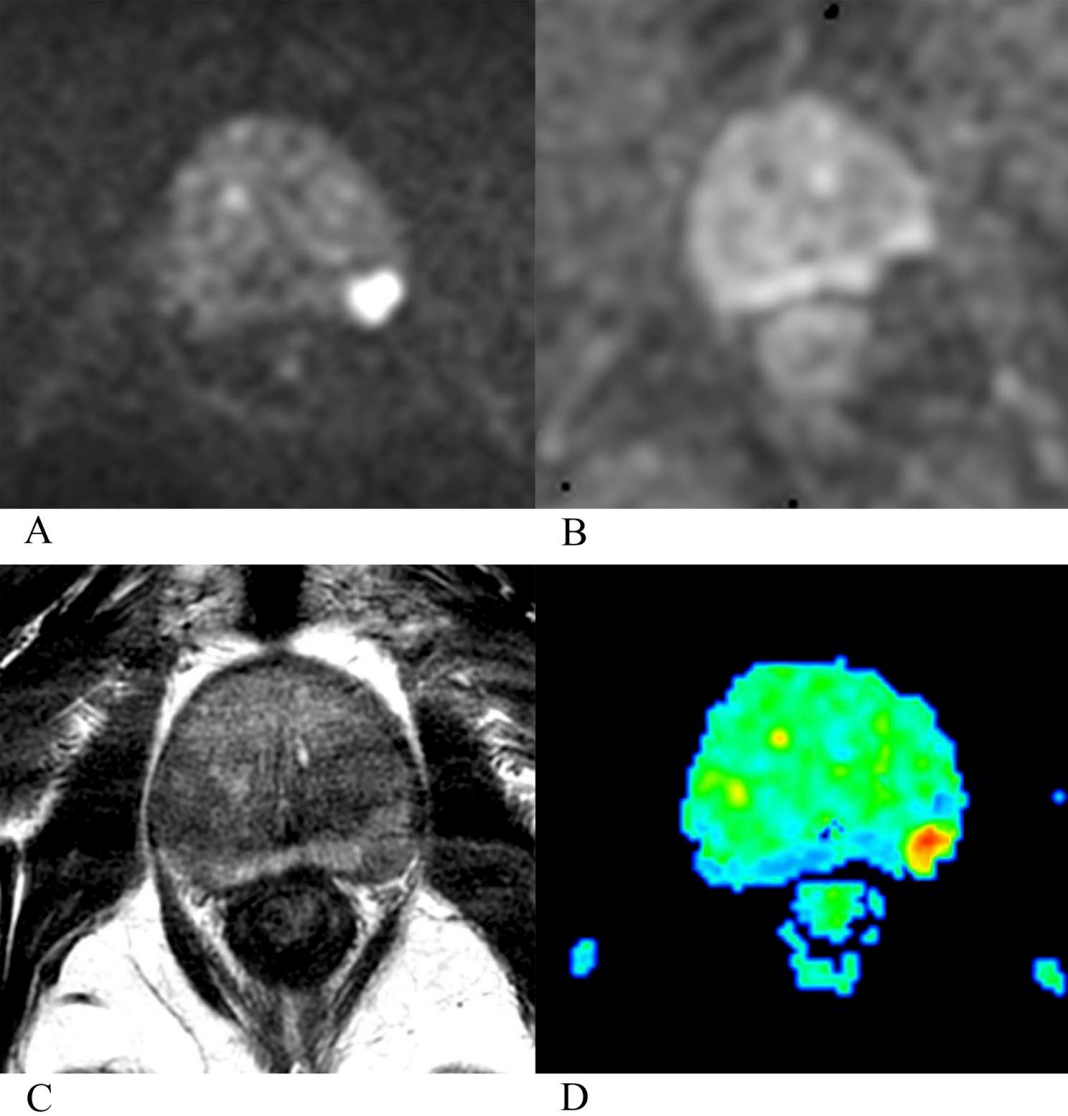
A 72-year-old International Society of Urological Pathology grade group 2 prostate cancer patient, with negative extraprostatic extension, negative perineural invasion, negative lymphovascular invasion and negative surgical margin, baseline T-PSA was 8.93 ng/ml, baseline F-PSA was 1.87 ng/ml, baseline F/T-PSA was 0.21. No biochemical recurrence was observed during the entire follow-up period. Preoperative multi-parametric MRI (**A**, diffusion weighted imaging; **B**, apparent diffusion coefficient map; **C**, T_2_-weighted imaging) showed that the PI-RADS 4 lesion was located in the left peripheral zone, and the mean kurtosis (MK) value was 0.571

**Fig. 3:**
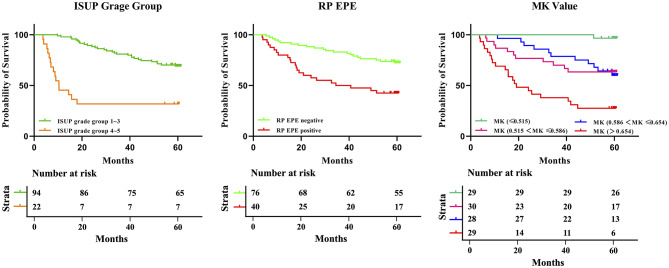
Kaplan-Meier curve of biochemical recurrence-free survival by International Society of Urological Pathology grade group (1 ~ 3 and 4 ~ 5), extraprostatic extension and radical prostatectomy surgical margin

Compared with non-BCR group, BCR group had a higher mean kurtosis (MK) value (0.548 ± 0.083 vs. 0.639 ± 0.065), had a lower mean diffusivity (MD) value (0.875 ± 0.123 × 10⁻³ mm²/s vs. 0.797 ± 0.117 × 10⁻³ mm²/s) and ADC value (1.057 ± 0.171 × 10⁻³ mm²/s vs. 0.968 ± 0.156 × 10⁻³ mm²/s). Results of independent sample *t*-test showed that the differences in MK (F = 4.840, t = − 6.231, *p* < 0.001), MD (F = 0.920, t = 3.341, *p* = 0.001) and ADC (F = 1.309, t = 2.795, *p* = 0.006) values between non-BCR and BCR groups were statistically significant. Details showed in Table 2; Figs. 1 and 2. Results of univariable Cox analysis showed that MK value (HR: 3.368; 95% CI: 2.221 ~ 5.105; *p* < 0.001), MD value (HR: 0.681; 95% CI: 0.535 ~ 0.868; *p* = 0.002) and ADC value (HR: 0.810; 95% CI: 0.677 ~ 0.968; *p* = 0.021) were the risk factors of BCR. The specific results provided in Table 3; Fig. 3. To more intuitively illustrate the predictive value of MK value for biochemical recurrence, we stratified patients according to quartiles of MK value and generated Kaplan–Meier curves for these MK-based groups. The corresponding statistical results are provided in the Supplementary Materials and Fig. 3.

### Multivariable analysis

The study built a parsimonious multivariable model integrating preoperative clinical/mpMRI characteristics with postoperative histopathological characteristics. Multicollinearity was assessed using the variance inflation factor (VIF), which revealed substantial collinearity among baseline T-PSA (VIF = 17.17), baseline F-PSA (VIF = 15.45), and baseline F/T-PSA (VIF = 12.11). To address this issue, we entered these variables separately into the multivariable Cox model and compared model stability and performance. The model including baseline T-PSA showed the best predictive performance, with a C-index of 0.887, outperforming models that included baseline F-PSA (C-index = 0.873) or baseline F/T-PSA (C-index = 0.843). Accordingly, baseline F-PSA and baseline F/T-PSA were excluded from subsequent ridge regression and internal cross-validation. After ridge penalization and internal cross-validation, the mean cross-validated C-index was 0.862. In the final model, baseline T-PSA (HR (95% CI), 10.579 (4.045–27.670); *p* < 0.001), MK (HR (95% CI), 3.034 (1.809–5.089); *p* < 0.001), ISUP grade group (HR (95% CI), 3.121 (1.352–7.209); *p* = 0.008), RP EPE (HR (95% CI), 2.219 (1.058–4.651); *p* = 0.035), and RP surgical margin (HR (95% CI), 2.816 (1.585–4.845); *p* = 0.025) were identified as independent risk factors for BCR. Details showed in Table 3.

## Discussion

By combining preoperative clinical and mpMRI characteristics with postoperative histopathology characteristics, the study identified the independent risk factors for BCR after RP for PCa, which may have important clinical significance for the individualized decision-making and follow-up planning of PCa patients after RP. In univariable analyses, baseline T-PSA, baseline F-PSA, baseline F/T-PSA, RP ISUP grade group 4 ~ 5, PR positive EPE, PR positive perineural invasion, RP positive surgical margin, RP positive lymphovascular invasion, MK value, MD value and ADC value were all significantly associated with BCR. In multivariable analysis, baseline F-PSA, RP ISUP grade group 4 ~ 5, RP positive EPE, RP positive surgical margin, and MK value remained independently associated with BCR, indicating that parameter MK value of DKI, is significantly associated with BCR after RP in PCa, which is similar to the markers in the traditional clinical-histopathological prediction models, and can be used as a preoperative imaging marker to predict BCR.

Clinically, DWI is one of the key sequences in prostate mpMRI and plays an essential role in PCa detection, guiding targeted biopsy, and staging [9, 18]. As an advanced sequence of conventional DWI, DKI quantifies the deviation of water diffusion from a Gaussian distribution, thereby enabling characterization of the complexity and heterogeneity of tissue microstructure [19]. Previous studies have demonstrated that DKI holds substantial potential for prostate cancer detection and assessment of tumor aggressiveness [11, 12, 20–23]. Moreover, prior evidence suggests that DKI outperforms conventional DWI in evaluating prostate cancer aggressiveness (e.g., Gleason pattern and reactive stromal hyperplasia) [10, 15], which is also reflected in our findings. In univariable Cox regression, ADC value (*p* = 0.021) was statistically significant, similar to parameters MK (*p* < 0.001) and MD (*p* = 0.002) values of DKI. However, in multivariable Cox regression, ADC value was no longer significant. These results indicate that DKI may provide additional information beyond conventional DWI for both assessing PCa aggressiveness and predicting BCR after RP, thereby offering clinically relevant insights to support decision-making. Notably, although both MK and MD values were significant in univariable analyses, only MK value remained significant in the multivariable model. This observation supports the hypothesis that kurtosis-based information may better capture recurrence-related microstructural alterations than diffusion-rate–based metrics alone, and it provides a theoretical rationale for incorporating DKI into preoperative risk stratification models.

Conversely, our data showed no significant association between PI-RADS 5 lesions and BCR in either univariable or multivariable analyses, which differs from some previous reports [24–26]. This discrepancy may be partly attributable to our relatively limited sample size. Given the widespread clinical adoption of PI-RADS, larger and preferably multicenter cohorts are needed to validate the relationship between PI-RADS category and oncologic outcomes.

Early models such as the D’Amico risk classification and more recent tools including the Memorial Sloan Kettering Cancer Center (MSKCC) nomogram, the Partin tables, and the Cancer of the Prostate Risk Assessment (CAPRA) score consistently indicate that presurgical clinical data (e.g., PSA) and postsurgical pathological features (e.g., ISUP grade group, EPE, and positive surgical margin) are independent predictors of BCR [27–32]. Our findings corroborate the prognostic relevance of these clinical and pathological variables. Notably, although both baseline F/T-PSA and T-PSA were significantly associated with BCR in univariable analyses, only baseline F/T-PSA remained an independent predictor in the multivariable model, suggesting superior stability and discriminative performance of baseline F/T-PSA over baseline T-PSA for BCR risk assessment. Contrary to previous studies [33, 34], RP perineural invasion and lymphovascular invasion did not retain independent significance after adjustment in our study. A plausible explanation is that these characteristics often reflect adverse pathological status closely linked to higher ISUP grade groups; thus, their effects may be largely captured by ISUP grade group within the model, diminishing their independent prognostic contribution.

This study has several limitations. Firstly, the study is a single-center analysis with a limited sample size; larger cohorts with external validation are needed to confirm the robustness and generalizability of our findings. Secondly, for DKI quantification (MK and MD values), some lesions were small and visible on only a single slice, which precluded reliable 3D delineation. To ensure methodological consistency, we therefore adopted a single-slice ROI placed on the largest axial section. While this approach improves comparability across the cohort, it may underestimate tumor heterogeneity and limit comprehensive characterization of overall tumor burden and microstructural features. Thirdly, while the quantitative imaging data (MK and MD values) we measured were not directly used to evaluate the pathological characteristics of the lesions, correlating it with pathological assessment results would undoubtedly enhance the interpretability of the findings and the reproducibility of the study. Fourthly, the model we developed to predict BCR after RP in patients with PCa was not compared with established prediction tools—such as the Memorial Sloan Kettering Cancer Center (MSKCC) nomogram [28], the Partin Table (32), or the University of California San Francisco (UCSF) Cancer of the Prostate Risk Assessment (CAPRA) score [29]—which undoubtedly weakens the clinical relevance of our study.

In conclusion, the study demonstrates that baseline F/T-PSA and histopathological characteristics—ISUP grade group, EPE, and surgical margin—were associated with BCR after RP. Importantly, parameter MK value of DKI remained an independent predictor of BCR in multivariable analysis. Integrating MK value with established clinicopathologic variables may provide quantitative imaging support for individualized postoperative surveillance.

## Supplementary Information

Below is the link to the electronic supplementary material.

Supplementary Material 1

## Data Availability

The data that support the findings of this study are available from the authors but restrictions apply to the availability of these data, which were used under license from the Second Affiliated Hospital of Dalian Medical University for the current study, and so are not publicly available. Data are, however, available from the authors upon reasonable request and with permission from the Second Affiliated Hospital of Dalian Medical University.

## Abbreviations

PCa: Prostate cancer
RP: Radical prostatectomy
PSA: Prostate-specific antigen
BCR: Biochemical recurrence
ISUP: International society of urological pathology
EPE: Extraprostatic extension
DWI: Diffusion-weighted imaging
DKI: Diffusion kurtosis imaging
MD: Mean diffusivity
MK: Value and mean kurtosis
ADC: Apparent diffusion coefficient
