# Supplementary material for: Prognostic features of biochemical recurrence of prostate cancer following radical prostatectomy based on diffusion kurtosis imaging
Source: Cancer Imaging. 2026 Jan 28;26:30. doi: 10.1186/s40644-026-00997-y (PMC12924370; doi:10.1186/s40644-026-00997-y)
Supplement: Supplementary file 1 — Supplementary Material 1 [file 40644_2026_997_MOESM1_ESM.docx]

| Table 1S Cox Regression Analysis for Biochemical Recurrence Based on MK value | | |
| --- | --- | --- |
| Variable | HR (95CI%) | p |
| MK ≤ 0.515 | Reference | |
| 0.515 < MK≤ 0.586 | 31.679 (3.558~282.065) | 0.018 |
| 0.586 < MK≤ 0.654 | 31.411 (3.154~312.813) | 0.003 |
| MK > 0.654 | 31.679 (3.558~282.065) | 0.002 |
| MK, mean kurtosis | | |
